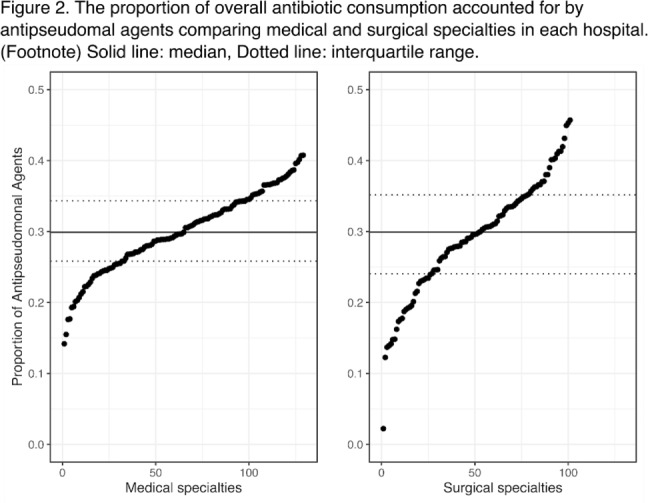# Hospital-level variation in the utilization of antipseudomonal antibiotics: A nationwide cross-sectional study at the VHA

**DOI:** 10.1017/ash.2022.92

**Published:** 2022-05-16

**Authors:** Shinya Hasegawa, Satoshi Kakiuchi, Daniel Livorsi, Eli Perencevich, Michihiko Goto

## Abstract

**Background:** Avoiding unnecessary antipseudomonal coverage is 1 of the most common targets for antibiotic stewardship programs (ASPs), but little is known about the magnitude of facility-level variation in antipseudomonal agent utilization. We aimed to describe the variability in the use of antipseudomonal agents across inpatient settings within a nationwide integrated healthcare system. **Method:** We analyzed the data from a retrospective cohort of patients who were admitted to acute-care hospitals within the VHA system in 2019. We defined antipseudomonal agents as systemic antibiotics with activity against wild-type *Pseudomonas aeruginosa*, and we evaluated overall and antipseudomonal antibiotic use among 129 hospitals, according to the agents described in the NHSN Antimicrobial Usage and Resistance Module. We calculated each hospital’s overall and antipseudomonal days of therapy (DOT) per 1,000 days present and the proportion of antipseudomonal agent usage among all antibiotics based on DOT at each hospital. Hospital-level variation was assessed by comparing the proportion of total antibiotic consumption accounted for by antipseudomonal agents. Associations between antipseudomonal proportions and overall antibiotic consumption were also assessed. **Results:** Among 129 VHA hospitals, the median DOT per 1,000 days present for all antibiotics was 434.4 (IQR, 371.9–487.1), and the median antipseudomonal DOT per 1,000 days present was 127.7 (IQR, 99.8–159.6). The median proportion of total antibiotic consumption accounted for by antipseudomonal agents was 30.0% (range, 14.9%–40.7%; IQR, 26.4%–34.4%) (Fig. 1). We detected only a weak correlation between overall antibiotic consumption and antipseudomonal proportion (Pearson correlation coefficient, 0.396), which suggests that hospitals with higher total antibiotic consumption were not necessarily using more antipseudomonal agents. In a stratified analysis, there was more prominent hospital-level variability in surgical specialties than medical specialties (Fig. 2). **Conclusions:** We detected high hospital-level variability in the consumption and proportion of antipseudomonal antibiotics among an integrated healthcare system. Although it is plausible that these variabilities originated from case-mix differences among hospitals, including differing rates of *P. aeruginosa* infections, it may also highlight opportunities for reducing antipseudomonal antibiotic utilization, especially among surgical specialties. Further studies are needed to evaluate the contribution of modifiable patient- and facility-level factors to this variability.

**Funding:** None

**Disclosures:** None

**Figure:**
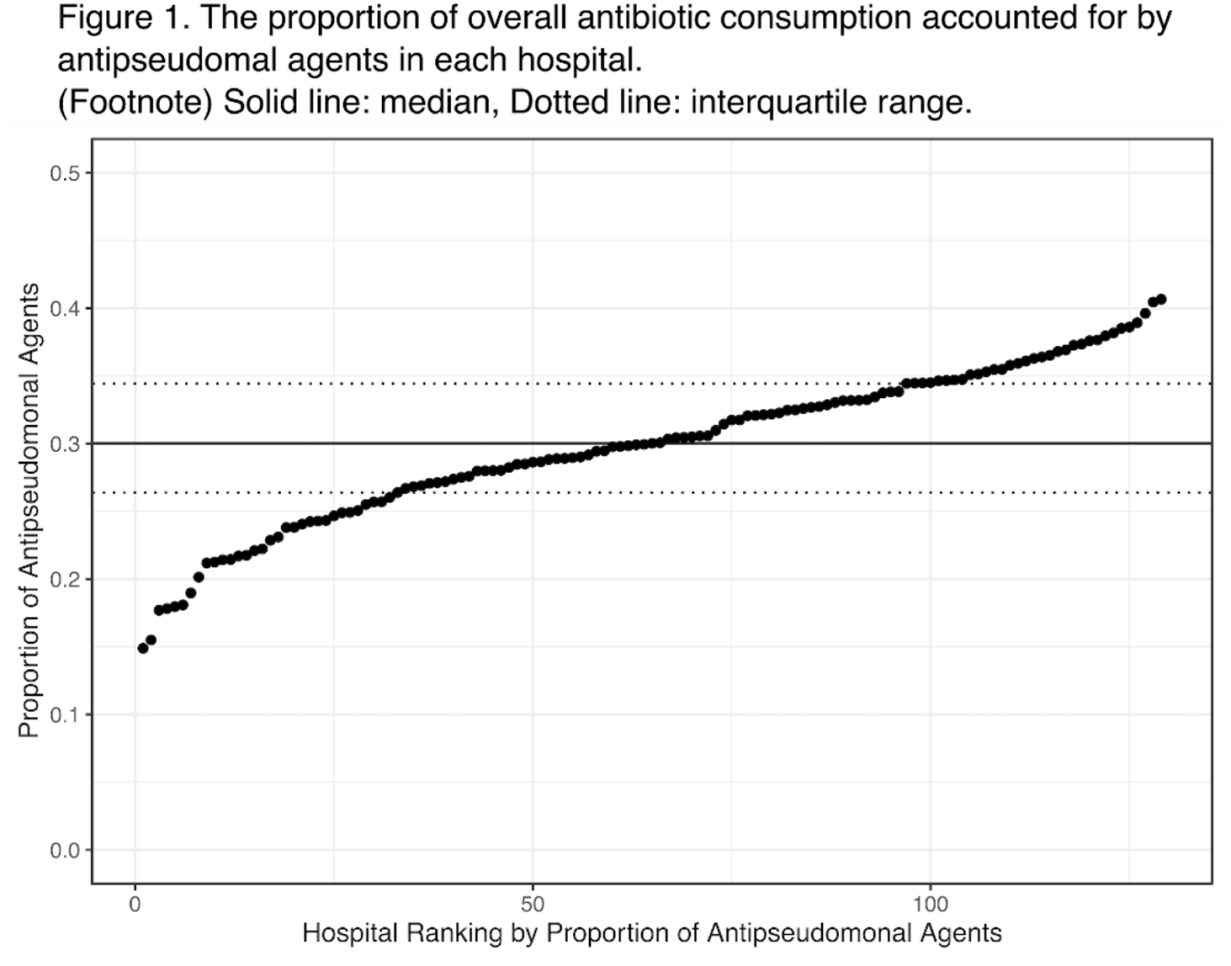

**Figure:**